# Electrical Conductivity in Graphite Foils Produced by Rolling and Pressing

**DOI:** 10.3390/ma17246153

**Published:** 2024-12-17

**Authors:** Nikolai S. Morozov, Vladimir A. Shulyak, Margarita G. Isaenkova, Olga A. Krymskaya, Vladimir A. Fesenko, Sergei N. Chebotarev, Victor V. Avdeev

**Affiliations:** 1 Department of Chemistry, Lomonosov Moscow State University, Moscow 119991, Russia; shulyak.v@unichimtek.ru (V.A.S.); chebotarev.s@inumit.ru (S.N.C.); avdeev@highp.chem.msu.ru (V.V.A.); 2 MEPhI (Moscow Engineering Physics Institute), National Research Nuclear University, Moscow 115409, Russia; mgisaenkova@mephi.ru (M.G.I.); oakrymskaya@mephi.ru (O.A.K.); fesenko.vlad@mail.ru (V.A.F.)

**Keywords:** flexible graphite foil, rolling, compacting, electrical conductivity, structural parameters, crystallographic texture, the Kearns texture parameters

## Abstract

In this research paper, the factors impacting electrical conductivity of the flexible graphite foils (GFs) produced by different forming processes, namely, either by rolling or pressing, were studied. The relationship between electrical conductivity and texture and structure that formed when producing the material was examined. Correlation was determined between the texture sharpness and anisotropy of electrical conductivity, as well as the extent of impact from the substructural characteristics on the properties’ values. Besides, it was demonstrated that the higher values of micro-strains, as well as the secondary phase substructure, reduced conductivity in foils. Electrical conductivity calculation was optimized for different directions in foils using the Kearns texture parameters and taking into consideration the foil structural characteristics.

## 1. Introduction

Flexible graphite foils (GFs), as the derivatives of natural graphite, and in addition to their considerable electrical and thermal conductivity [1,2], possess high chemical and thermal resistance [3]. Such properties, together with flexibility and mechanical strength, broaden even further the already large range of application for the graphite materials in various key industries. GFs are applied in manufacturing of graphite seals [4,5] and graphite gaskets [6,7], in the oil-and-gas industry, in the nuclear and thermal power engineering, in manufacturing of the climate panels to ensure retention of temperatures in rooms, or in separate pieces of machinery, to produce heat-spreading structural parts required for cooling the heaters in utilities [8] and the energy storage systems [9], as well as in the conductive elements (positive electrode) of the lithium-ion batteries [10] and supercapacitors [11,12].

Normally, production of GFs involves processing flake graphite with strong acids to obtain graphite intercalation compounds (GIC) [13], followed by hydrolysis and synthesis of the thermally exfoliated graphite (TEG) [14]. Further, the thermally exfoliated graphite undergoes forming processes to achieve the desired density of GF. At that, the high-density graphite foils (with density > 1.6 g/cm^3^) demonstrate higher indices of electrical conductivity owing to the close-packing layers of the TEG [15,16].

The forming process induces various meso- and nano-structural changes in graphite foils [17]. In addition to the in-plane alignment of the TEG particles and deformation of transverse compression, the appropriate or crystallographically controlled re-orientation of the nano-crystallites takes place in such a way that some crystallographic texture is formed [18]. Such structural modifications are meaningful when determining the extent of electrical conductivity for the foils. The crystallographic texture of the rolled graphite foils is the important constituent in defining the conductive properties and their anisotropy [19,20]. The preferred orientation of the nano-crystallites can be described through determining the Kearns texture parameters, which were originally elaborated for the needs of analyzing the anisotropy properties in zirconium alloys [21,22], yet equally applicable to the graphite materials. Besides, the anisotropic nature of GF impacts its performance in terms of methods of application. The ability to control the conductive properties of GFs in the course of their production, using the controlled pressure-forming techniques, offers significant advantages for their application in the advanced technologies, comprising flexible electronics [23], batteries [24,25,26], and electromagnetic interference shielding [27].

Electrical conductivity of the graphite foils has been studied sufficiently [28,29,30]. Still, in our opinion, the relation between the change in electrical conductivity and the method of forming processes of the TEG, i.e., between the structural and textural transformations under the mechanical action, has not been described in detail.

Consequently, the objective of this research paper is to determine the electrical conductivity characteristics for graphite foils produced by cold rolling and pressing, as well as to determine the impact of the structural and textural parameters on the final properties of the material.

## 2. Materials and Methods

### 2.1. Production of the Graphite Foil

**Figure 1:**
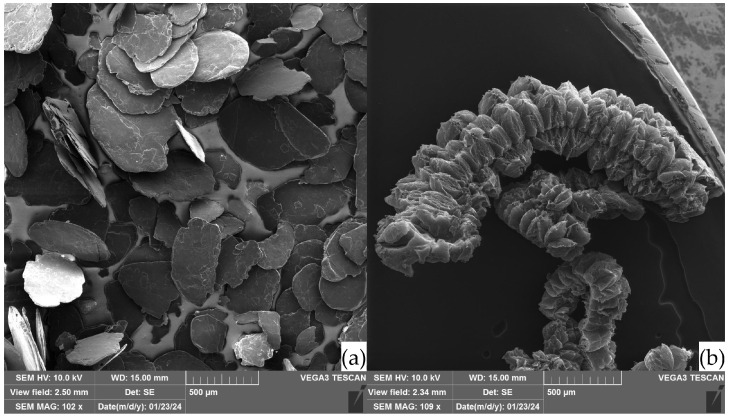
Images of natural graphite (**a**) and TEG (**b**).

Graphite foil of 1.2 mm thick was produced from the natural graphite (Taiginsky Mining and Processing Plant LLC, Kyshtym, Russia) (Figure 1a), which was used for synthesis of the oxidized graphite (OG), followed by subsequent thermal decomposition of the latter and formation of the thermally exfoliated graphite (TEG; Figure 1b) [31,32]. Over the course of our research study, we applied intercalation of graphite with nitric acid, as per the nitrate method for generating the graphite intercalation compounds (IGC) [33,34,35].

The process of producing the graphite foil samples comprised several stages. Initially, natural graphite was processed with the fuming nitric acid (Novomoskovska aktsionerna kompaniia “Azot”, Novomoskovsk, Russia) at 1:0.8 by mass, thus producing GIS. Then, GIS was hydrolyzed and dried, and at the end, the OG adduct was generated. At the next stage, OG was heat-processed at the temperature of 900 °C, which resulted in its foaming [36,37].

**Figure 2:**
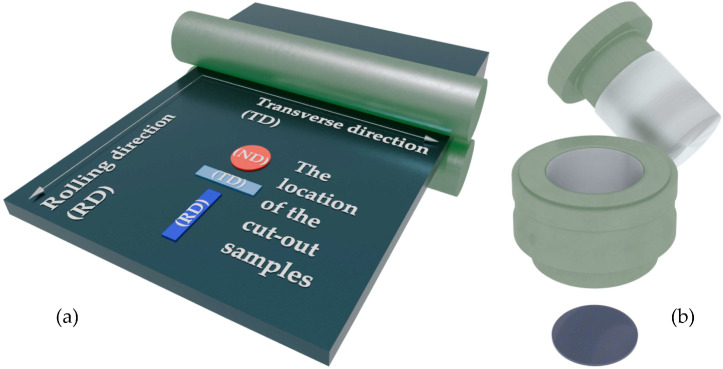
Algorithm for generating the rolled (**a**) and pressed (**b**) samples.

Once produced, the TEG underwent two methods of forming processing: rolling and uniaxial pressing. Under the rolling process, TEG passed through the rolling mill rollers (MTI Corp, Richmond, VA, USA), was compacted, and GF was produced (Figure 2a). As for the uniaxial pressing, TEG was positioned into a mold and exposed to pressure, which, in fact, led to formation of graphite foil (Figure 2b).

Next, the samples were prepared for different studies (Table 1). The cut-up sketch for the samples from the rolled foil is presented in Figure 2a. Specific electrical conductivity of graphite foils was measured in the rolling (RD), transverse (TD), and normal (ND) directions. In order to measure the electrical conductivity of the pressed GF, several samples were processed in the same way. To measure the electrical conductivity, the pressed graphite foil samples were carefully cut down to the shape of a square. The rolling direction (RD) was set at random for them, and the transverse direction (TD) was chosen to be 90° relative to the RD.

For each sample, the physical dimensions and density values were taken. The thickness of the samples was measured with a micrometer (Machinery & tools company limited, Jiangsu, China) (misalignment < 4 μm), while length and width (and diameter for the pressed samples) were measured with a caliper (ASIMETO Germany GmbH, Niedernhall, Germany) (misalignment < 10 μm). The mass of the samples was measured using a precision scale Shimadzu AX-200 (SHIMADZU CORPORATION, Kyoto, Japan) (misalignment < 0.001 g). The utmost misalignment of the samples’ physical dimensions reached <2%, with density at 2%.

### 2.2. Determination of Electrical Conductivity of the Graphite Foil

For the purposes of measuring specific electrical conductivity, the modified van der Pauw four-point probe method was chosen (the van der Pauw method) [38,39,40,41]. The source of the direct current was GPD-73303D, and the voltmeter was GDM-78255A (both devices are by the GW Instek brand, Taiwan, China). The elementary graphite foil sample of 50 ± 1 mm in length and 10.0 ± 0.5 mm in width was positioned on two pairs of platinum contacts at 25 °C. The first pair of contacts supplied the direct current to the sample, and the second one measured the voltage. Measuring began by plotting the voltampere characteristic within the current range, from 0.1 to 0.5 A. The arithmetic mean value of the voltage was taken to compensate for the Seebeck effect. Specific resistance of the sample was calculated using Equation (1), and specific conductivity using Equation (2):

(1) $$\rho =\frac{R\cdot (b\cdot h)}{l}$$

(2) σ=1/ρ

where *R* is resistance, *b* is width, *h* is thickness, and *l* is length between the contacts.

### 2.3. Scanning Electron Microscopy Examination of Graphite

The microstructure of the graphite foils (GFs) was analyzed using scanning electron microscopy (SEM) TESCAN VEGA 3 (TESCAN, Brno, Czech Republic). The instrument was equipped with an electron gun utilizing a lanthanum hexaboride cathode. The electron beam was operated at an accelerating voltage of 10 kV. Secondary electron (SE) detection was employed for imaging. SEM micrographs were captured with a field of view of 2000 × 2000 μm.

### 2.4. Measuring the Nano-Crystallite Sizes

The study of the structural characteristics of GFs was carried out using the X-ray Rigaku Ultima IV diffractometer (Tokyo, Japan), with the Bragg–Brentano focusing geometry. The CuKα radiation with a wavelength of 0.15418 nm was applied. The XRD patterns for GF samples were recorded in the θ/2θ mode at the angles of 2θ = 20–90° (step: 0.02°, speed: 1°/min). Considering the instrumental broadening, the XRD pattern was recorded for the LaB_6_ standard sample.

For the purposes of assessing the structural changes in graphite, the characteristics of the XRD patterns were studied. Periods for the graphite crystal lattice, *a* and *c*, were determined. The full width at half-maximum (FWHM) was calculated, employing the Williamson–Hall method [42,43], which enabled accounting for the impact from the micro-strains when studying the nano-crystallites’ parameters (Equations (3)–(5)):

(3) $$FWHM=\beta_{phys}+\beta_{ins}=\beta_{L}+\beta_{\varepsilon }+\beta_{ins};$$

(4) $$\beta_{phys}=\frac{0.9\cdot \lambda }{L_{c}\cdot \cos\theta }+4\cdot \varepsilon_{micro}\cdot \tan\theta ;$$

(5) $$\beta_{phys}\cdot \cos\theta =\frac{0.9\cdot \lambda }{L_{c}}+4\cdot \varepsilon_{micro}\cdot \sin\theta ;$$

where $\beta_{phys}$ is physical broadening of the reflection caused by the substructural features of the material, $\beta_{ins}$ is input from the instrumental broadening caused by the diffractometer’s characteristics, $\beta_{L}$ is input from the broadening owing to the fine dispersion of the nano-crystallites, $\beta_{\varepsilon }$ is broadening caused by the micro-strains being present, λ is the wavelength of the X-ray radiation, $L_{c}$ is the size of the nano-crystallites along the parameter c axis of the graphite crystal lattice, θ is the Bragg’s angle of the X-ray reflection, and $\varepsilon_{micro}$ are micro-strains within the nano-crystallites.

The Williamson–Hall method involves plotting the dependence (Equation (5)) on 4·sin⁡θ, which is approximated by the linear function in order to determine micro-strains and the nano-crystallite parameters. Such method of calculation enables minimization, to a significant extent, of the error of the estimate relative to the outcome, as obtained using the Scherrer equation [44,45].

### 2.5. Misorientation of Nano-Crystallites

Application of the X-ray technique for recording the rocking curves, to determine the misorientation angles, enabled conducting a more detailed study of the nano-crystallites’ structural characteristics, as well as their impact on the material physical properties. The misorientation angles (MA) of the GF nano-crystallites were defined using the Rigaku Ultima IV diffractometer, as per the rocking curves of reflection (0006) in hexagonalclose-packed (hcp) graphite. The studies were carried out using the CuKα radiation in the θ mode and at the fixed angle of 2θ, while scanning was carried out at θ angles ranging from 10° to 80°. The obtained data enabled determination of the full width at half-maximum (FWHM) for the rocking curves, the value of which was the misorientation angle value.

### 2.6. Identification of the Preferential Crystallographic Orientation of Nano-Crystallites and Calculation of the GF Electrical Conductivity

For the purposes of the crystallographic texture analysis, we used the D8 Discover X-ray diffractometer (Bruker AXS, Billerica, MA, USA) equipped with the POLYCAP poly-capillary optics system (to form a small cross-section beam of high intensity and divergence lower than 0.2°), and four pole figures (PFs), (0002), {10$\bar{1}$1}, {10$\bar{1}$3}, and {11$\bar{2}2\},$ were registered. 

Calculation of the Kerns texture parameters (the *f* parameters), which are the integrated volume fractions of the projections of the basal axes [0001] on the selected direction within the sample, was carried out involving the recorded PF (0001) obtained with the tilt angle up to 80°, as per the technique described in [22,46].

One of the most common methods for the anisotropy calculation of some properties (*PP*) in the materials with the hcp structure is averaging the crystallites’ properties (single crystals) using *f* parameters (Equation (6)) [47,48]:

(6) $${PP}_{\psi }={PP}_{c}\cdot \sum_{i}V_{i}{cos}^{2}\psi_{i}+{PP}_{a}\cdot \sum_{i}V_{i}(1-{cos}^{2}\psi_{i})=f_{\psi }\cdot {PP}_{c}+(1-f_{\psi })\cdot {PP}_{a},$$

where PP*c* and PP*a* are the property values along the axes of the hexagonal crystal lattices *c* and *a*, respectively, *Vi* is the volume fraction of the crystallites in the direction under study, and ψ_*i*_ is the deviation angle of the selected direction of the *i*-th grain from the basal axis, i.e., from the c axis.

Given the fact that this formula does not account for interaction of the grains, nor for any potential impact of the structural factors, the research paper in [17] defined the coefficient that comprises the meso- and nano-structural characteristics to enable calculation of the GF electrical conductivity (Equation (7)):

(7) $$\sigma_{\psi }=n_{\sigma }\cdot \left[f_{\psi }\cdot \sigma_{c}+\left(1-f_{\psi }\right)\cdot \sigma_{a}\right]=\frac{1}{8}\cos(MA)\cdot \frac{d}{d_{Gr}}\cdot \left[f_{\psi }\cdot \sigma_{c}+\left(1-f_{\psi }\right)\cdot \sigma_{a}\right],$$

where σ*_c_* = 10 kS/m, σ*_a_* = 2500 kS/m [49], *d* is the density of the studied GF, and *d_Gr_* is the X-ray density of graphite.

## 3. Results and Discussion

### 3.1. Experimental Value of Electrical Conductivity for Graphite Foils

**Figure 3:**
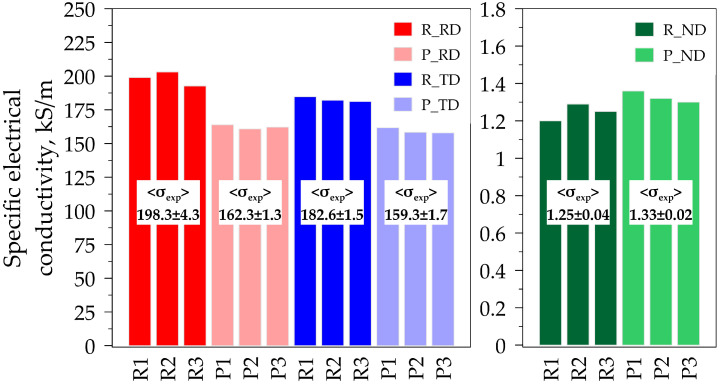
Specific electrical conductivity values ($\sigma_{exp}$) for graphite foils produced by rolling and pressing in different directions.

The studies were carried out using the three samples of graphite foils produced by rolling and pressing. Electrical conductivity ($\sigma_{exp}$) was measured in three directions: along the rolling direction (RD), across the transverse direction (TD), and in the normal direction (ND). The outcome is shown in Figure 3.

Based on the outcome of the analysis for the rolled graphite foils, it was possible to find certain significant anisotropy in their electrical conductivity. The values of electrical conductivity in the rolling direction (RD) and in the transverse direction (TD) were much higher than those in the normal direction (ND). This can be explained by specifics of the graphite structure: alongside the graphene layer’s plane (the rolling plane), sufficiently high conductivity of the electric current was observed, rather than along the plane perpendicular to the basal one. The average values of electrical conductivity in the RD and TD for the rolled foils were 198.3 kS/m and 182.6 kS/m, respectively, while in the ND, this value dropped to 1.25 kS/m. For the pressed foils, electrical conductivity in the RD and TD was 162.3 kS/m and 159.3 kS/m, respectively, and in the ND it was 1.33 kS/m.

It needs to be mentioned that the deviations in the resulting values of electrical conductivity measurements for all samples were insignificant. This points to the reproducibility and accuracy of the measurements. The lower values of standard deviations (4.3 kS/m for the RD rolled foils, and 2.1 kS/m for the RD pressed foils) proved the stability of the obtained data.

The obtained outcome implied significant anisotropy of the properties in graphite foils. Anisotropy of electrical conductivity occurs because in the graphite crystal structure basal planes, which are graphene layers, there are strong covalent bonds, thus, the chances of an electron penetrating through the high electron density of the covalent bond are very low. At the same time, the layers are connected to each other by metallic bonds with one free electron, and this determines the high values of conductivity along the basal planes. This, in fact, is indicative of the low values of electrical conductivity in the normal direction (ND) and the high ones in the RD and TD directions.

It needs to be mentioned that the technique used to produce foils impacted significantly on their electrical conductivity. The rolled foils demonstrated higher conductivity values than the pressed foils. Nonetheless, such processing caused anisotropy in the rolling plane. Pressing led to an increase in the isotropic values for conductive properties in the rolling plane. Primarily, this can be justified by the fact that the rolling process contributed to the increasing sharpness of the crystallographic texture, which increased conductivity. On the other hand, the pressed foils showcased lower conductivity values in the RD and TD. This may occur because of the fact that the pressing process contributed to orientation dispersion of the basal exponents in vicinity of the ND.

The second factor that may impact conductivity is porosity and defectiveness of the crystal structure. According to the GF density assessment, the total porosity value for the samples was the same, and this does not explain such a difference in properties. Another explanation may constitute the different nature of the pores or defects’ distribution within the material (meso-structure), as well as the structural state of the material (microstructure).

### 3.2. Phase Composition and Structural Characteristics of GF

**Figure 4:**
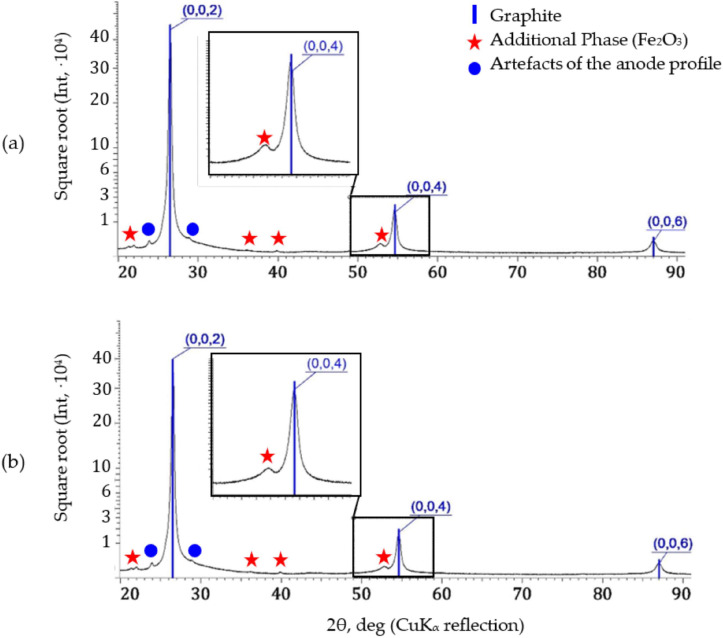
The XRD patterns of the rolled (**a**) and pressed (**b**) samples.

Figure 4 provides examples of the fragments of XRD patterns for the rolled and pressed samples, because diffraction patterns of the three samples (R1, R2, and R3 and P1, P2, and P3) were virtually the same within the same batch. In order to identify the additional phases, the intensity values were provided in the square root scale. With reference to Figure 4, it is evident that next to the graphite’s reflection (0004), there is a line of an additional phase, and, apparently, this is iron oxide Fe_2_O_3_, the content of which is lower than 1%. The ash content of the material used to produce foils for the purposes of writing this article was presented in our previous study [17]. Judging by the nature of the XRD patterns, the additional phase had a preferential orientation as well; however, for the needs of a comprehensive description of its structural and textural characteristics, only single reflection was not enough. In such a case, this peak was approximated by the Pseudo–Voigt function only. The structure of the additional phase was analyzed using the ${FWHM}_{AP}\;technique$ (*AP* is the additional phase) and by the ratio of the integral intensities of the Fe_2_O_3_ peak to the nearest graphite peak (0004): *I_AP_*/*I*_(0004)_.

On the grounds of the XRD analysis patterns, evaluation of the nano-crystallite and micro-strain parameters for the graphite foils produced by either rolling or pressing took place. Their values were of great interest to enable understanding of the structural characteristics of the studied materials, as well as their impact on electrical conductivity.

Table 2 below summarizes the data on the structural characteristics of the GF samples: the periods of the crystal lattice, *a* and *c*, as well as the sizes of nano-crystallites along the *c* axis (*L_c_*) and micro-strains ($\varepsilon_{Lc}$). The crystal lattice periods *a* and *c* were determined owing to the presence in the XRD patterns, not just the predominant reflections (000*l*), which are characteristic of the sharp basal texture, but also the low-intensity peaks from the prismatic planes’ reflections. The data in Table 2 indicate that the values of the parameter *c* for the rolled (R1, R2, and R3) and pressed (P1, P2, and P3) samples revealed no differences. However, the period *a* for the pressed foils was lower.

Since the values of the parameter *c* for the crystal lattice of the rolled and pressed materials were the same, the lower level of micro-strains was witnessed in the rolled samples, namely, the defectiveness of the sample material lattice was lower. The property of conductivity in materials was impacted to a great extent by the crystal structure perfection. Consequently, the values of electrical conductivity in the basal plane of the rolled foils were higher, and they were lower in the normal direction because of the electric charge transfer along the ND (perpendicular to the basal planes), owing to presence of the structural defects.

The nano-crystallite sizes (*L_c_*) in the rolled samples varied from 33.8 nm to 39.6 nm, while for the pressed ones, their range was from 36.5 nm to 40.8 nm. The values for the rolled and pressed GF samples were virtually similar. This points to the fact that the type of forming process did not impact the final size of nano-crystallites along the hexagonal crystal lattice *c* direction. It was not possible to estimate the nano-crystallite sizes for graphite foils in another (*a*) direction of the crystal lattice. In the spectra obtained for the rolling plane, the intensity of additional reflections, other than (000*l*), owing to the high texturing of the studied foils, was too low to correctly apply the method for calculating the crystallite size while taking into account the micro-strain. Sample processing of the GF cross-section was also impossible owing to the low strength of graphite itself. Therefore, the sample processing did not take place within the framework of this research study.

Micro-strains in the rolled GF samples revealed lower values (0.42–0.56%) when compared to the pressed ones (0.51–0.59%). The higher micro-distortions may point to the presence of a greater number of defects present in the graphite structure. This impacts the electrical conductivity of the material, reducing it owing to the increased scattering of the charge carriers.

Another factor that may impact the GF electrical conductivity is the presence of the impurities. On the grounds of the structural characteristics analysis for the impurity phase, it follows that higher ${FWHM}_{AP}$ values were witnessed in the pressed samples when compared to the rolled GF. This points to its finely dispersed structure and the increased levels of micro-distortions. Consequently, the finely dispersed impurity phase, owing to presence of a large number of the crystallite boundaries, may impact electrical conductivity in the pressed foils in a negative way.

Furthermore, the outcome enabled concluding that the additional phase for the pressed samples had a greater ${FWHM}_{AP}$ value, namely, it was finely dispersed and had more micro-strains. This may additionally impact the electrical conductivity of the pressed samples.

Another structural characteristic that can affect the final properties comprises the preferential orientation of the nano-crystallites relative to the external directions (RD, TD, and ND) in GF, namely, their crystallographic texture.

### 3.3. Crystallographic Texture of GF and the Calculated Values of Electrical Conductivity

**Figure 5:**
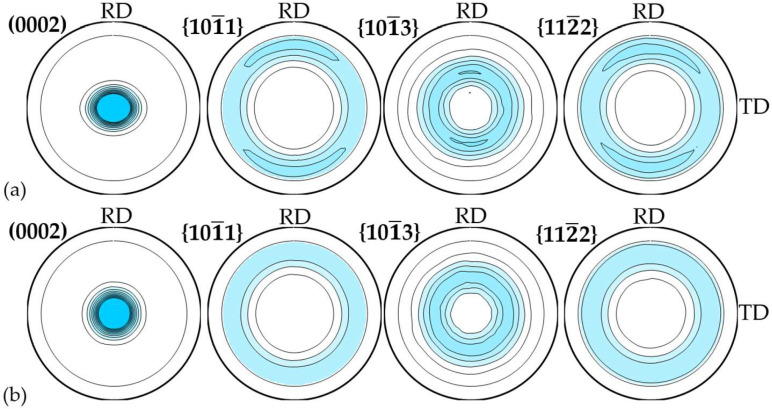
Experimental PFs of the studied rolled (**a**) and pressed (**b**) GF samples.

The examples of experimental PFs in the rolled and pressed GF materials are shown in Figure 5. It is noteworthy that the appearance of the PFs for samples within the same batch was virtually the same.

Judging by the obtained experimental PFs (Figure 5), it is obvious that the samples featured a preferential orientation of the basal planes (0001) parallel to the GF plane, or to the exponents [0001]‖ND.

The texture of the rolled foils was sharper (more expressed) when compared to pressed ones, and this was proven by the quantitative parameters (Table 3). Some insignificant asymmetry of the texture maximum could be equally noted for the rolled foils: In the section of ND–TD, it had a larger half-width when compared to ND–RD. The asymmetry of the texture maximum impacted the character of the PFs {10$\bar{1}$1}, {10$\bar{1}$3}, and {11$\bar{2}$2}, which revealed axial distribution in the GF plane, yet with a slight increase in the pole density in ND–RD. As for the pressed samples, the maximum was symmetrical, and the texture was of an axial type, which was due to the axisymmetric manufacturing scheme (uniaxial compression).

The analysis of the values of the misorientation angles of the basal planes (0001) for the rolled and pressed GF samples (Table 3) acted as evidence of the structural heterogeneity in the samples. The rolled foils had smaller misorientation angles, namely, (24.4 ± 0.1)° when compared to the basal exponents in the rolling direction vs. the angles in the transverse direction, (27.3 ± 0.2)°. This correlated with the PF data (maximal asymmetry).

Pressing of GF led to the appearance of large misorientation angles in the nano-crystallites both in the rolling direction (RD) and in the transverse direction (TD). The average value of the misorientation angles for the pressed foils was (27.6 ± 0.7)° in the pressed sample plane.

The foils with higher values of misorientation angles featured lower electrical conductivity, which correlated with the texture parameters determined by the PFs: along the directions with a more pronounced orientation of the basal axes <0001> (for example, *f*_RD_ = *f*_TD_ = 0.099 for pressed samples), the electrical conductivity decreased. The same occurred along different directions in the plane of the rolled samples: along the RD with a smaller MA (24.4°) and *f* parameter (*f*_RD_ = 0.083), higher σ values of electrical conductivity were observed when compared to the TD. At the same time, the lowest electrical conductivity was observed along the ND of the rolled samples, with the highest *f*_ND_ = 0.820.

Thus, for samples with more pronounced orientation of the basal axes along the ND (higher σ values of *f*_ND_ and lower MA), the electrical conductivity in the foil plane along the RD/TD increased significantly.

Correlations between the misorientation angles and the extent of micro-distortions in the materials were determined. The higher misorientation angles, revealed in the pressed samples, could represent a great number of defects in the structure, which was further proven by the greater micro-strains in them.

The electrical conductivity values ($\sigma_{calc}$) for three directions in the GF samples, as calculated using the integral texture parameters and taking into account the structural coefficients (Equation (4)), are presented in Table 4 along with the deviations from the experimental values, calculated as $\left|\sigma_{exp}-\sigma_{calc}\right|$. As the RD was not available for the pressed samples, calculations along two directions in the GF plane, located at an angle of 90° relative to each other, were provided for them. When calculating $\sigma_{calc}$ along the ND, the MA value was taken at 85 ° on the grounds of the fact that absence of the basal exponents in this direction was equivalent to the fact that the (0002) line featured almost complete blurring of the maximum, the half-width of which reached 90°. Still, scattering of the texture led to the fact that very weak reflections were observed, which could not be calculated.

The obtained data showed that the $\sigma_{calc}$ values in the GF plane (RD and TD) were closer to the experimental values for the rolled samples, yet for the pressed samples they were largely overestimated, which correlated with the structural data: presence of defects in the pressed samples was higher, and which additionally reduced electrical conductivity. In this regard, Equation (7) needs to be modified, taking into consideration the real substructures of the samples. Additionally, increased deviations for TD were observed in the rolled samples, which also points to the need to perform the coefficient adjustment.

Anisotropy of the GF electrical conductivity is shown in Table 5. In accordance with its indicators, it was obvious that the calculated values of the coefficient for the ND differed approximately three-fold. This also showcased the impact from the structural factors. It is known from the reference sources [49] that, for instance, anisotropy of the crystallite sizes can be found in GF. Thus, Lc can be several (two or three) times lower when compared to La, namely, to the size of the crystallites along the prismatic exponents, which, in fact, would equally impact electrical conductivity. Besides, owing to the very nature of graphite, electrical conductivity, perpendicular to the basal planes, can be justified by a pattern different from the one that occurs along the basal planes, namely, it can have a great impact due to the number of crystallite boundaries of graphite itself, and impurities within it.

It is also worth mentioning the lattice spacing *a*, which, in spite of the lower accuracy of determination, nonetheless differed for the pressed and rolled samples. It is thought that, when atoms are brought closer together, namely, at a lower lattice spacing, the amplitude of oscillations decreased owing to strengthening of the bonds. This also led to a decrease in mobility of the charge carriers and, consequently, to a decrease in electrical conductivity itself. This additionally proved the lower values of σ*_exp_* in the RD and TD for the pressed samples when compared to the rolled ones.

Besides, it is noteworthy that when compared to the theoretical values for graphite (*a*_theor_ = 246.2 and *c*_theor_ = 671.1 pm), both of the lattice parameters were slightly increased. This may point to the presence of single defects, which also impacts electrical conductivity. On the other hand, an accurate calculation requires supportive research studies, since the lattice parameters contribute to various components of conductivity.

For the needs of understanding the extent of the impact of micro-strains, the sizes of nano-crystallites, or their anisotropy, it is also required to carry out measurements in various directions. Still, the intensity of reflections, by which *a* was estimated, is insufficient to determine the structural characteristics. In compliance with the above, treatment of the corresponding samples at this stage does not appear possible.

## 4. Conclusions

Over the course of this research study on the conductivity of graphite foils (GFs) of the same thickness and density, and produced by rolling and pressing, differences in the values and anisotropy of their specific electrical conductivity, dependent on the technological processing, were revealed.

It was demonstrated that rolling of GF resulted in the formation of a sharper texture when compared to pressing and, accordingly, in increased values of specific electrical conductivity and its anisotropy relative to the exponent vs. the foil plane. With this, anisotropy of the texture maximum and deviation from the isotropic distribution of electrical conductivity along the foil plane were revealed in the rolled GF when compared to the pressed samples. For these, the axisymmetric manufacturing scheme ensured formation of the symmetric maximum and the same σ*_exp_* values in the RD and TD.

It was proven that the elements of the meso-structure (porosity and impurities) and substructure (disorientation of nano-crystallites, parameters, and distortion of the crystal lattice) had an impact on the absolute value of the specific electrical conductivity. In the pressed samples, it was lower owing to the increased distortion of the crystal structure, which was characterized by micro-strain and dispersion of both phases.

Calculation of the specific conductivity values on the basis of the Kerns texture parameters and the properties of the monocrystal pointed to acceptable congruency with the experimental data. Thus, it is required to expand the theoretical aspects of the conductivity mechanisms in graphite foils in order to account more accurately for the impact of the substructural parameters.

Future research directions include refining theoretical models of electrical conductivity to incorporate nanoscale structural influences, such as lattice distortions and nano-crystallite misorientation, and further investigating the relationship between these features and the physical, mechanical, electrical, and thermal properties of graphite foils.

## Figures and Tables

**Table 1:** Names of the manufactured GF samples.

Processing	Sample Name	Type of the Study
Rolling	R1, R2, R3	Study of the structural and textural characteristics
	R1_RD, R2_RD, R3_RD	Measuring electrical conductivity in different directions
	R1_TD, R2_TD, R3_TD	
	R1_ND, R2_TD, R3_TD	
Pressing	P1, P2, P3	Study of the structural and textural characteristics
	P1_RD, P2_RD, P3_RD	Measuring electrical conductivity in different directions
	P1_TD, P2_TD, P3_TD	
	P1_ND, P2_ND, P3_ND	

**Table 2:** Structural characteristics of the studied GF samples.

Sample	Graphite				Additional Phase	
	*a*, pm	*s*, pm	*L_c_*, nm	$\varepsilon_{Lc}$, %	${FWHM}_{AP}$, °(2θ)	*I_AP_*/*I*_(0004)_
R1	247.36	672.27	38.5	0.55	1.05	0.118
R2	247.47	672.29	33.8	0.42	1.06	0.119
R3	247.48	672.29	39.6	0.56	1.02	0.113
<R>	247.44 ± 0.07	672.28 ± 0.01	37.3 ± 3.1	0.51 ± 0.08	1.04 ± 0.02	0.117 ± 0.003
P1	247.08	672.29	36.5	0.51	1.14	0.112
P2	247.16	672.25	38.5	0.56	1.11	0.110
P3	246.72	672.29	40.8	0.59	1.13	0.107
<P>	246.99 ± 0.23	672.28 ± 0.02	38.6 ± 2.2	0.55 ± 0.04	1.13 ± 0.02	0.110 ± 0.003

**Table 3:** Texture characteristics of the studied GF.

Sample	Misorientation Angle (MA), °		Integral Texture *f* Parameter		
	‖RD	⊥RD	*f*_ND_	*f*_TD_	*f*_RD_
R1	24.6 ± 0.4	27.5 ± 0.5	0.818	0.084	0.098
R2	24.3 ± 0.4	27.1 ± 0.5	0.822	0.082	0.096
R3	24.3 ± 0.4	27.2 ± 0.5	0.819	0.084	0.097
<R>	24.4 ± 0.1	27.3 ± 0.2	0.820 ± 0.002	0.083 ± 0.001	0.097 ± 0.001
P1	28.1 ± 0.5	27.7 ± 0.5	0.807	0.097	0.097
P2	26.7 ± 0.5	26.8 ± 0.5	0.809	0.095	0.096
P3	28.1 ± 0.6	28.4 ± 0.6	0.790	0.105	0.105
<P>	27.6 ± 0.7	27.6 ± 0.7	0.802 ± 0.009	0.099 ± 0.004	0.099 ± 0.004

**Table 4:** The calculated values of the GF electrical conductivity.

Sample No.	$\sigma_{calc}$ for the Rolled GF (R), kS/m			$\sigma_{calc}$ for the Pressed GF (P), kS/m		
	RD	TD	ND	RD	TD	ND
1	204.9	196.8	3.9	196.0	196.7	4.2
2	205.8	198.0	3.9	198.9	198.5	4.2
3	205.4	197.6	4.0	194.2	193.7	4.6
Mean values	205.4 ± 0.4	197.5 ± 0.5	3.9 ± 0.1	196.3 ± 2.1		4.3 ± 0.2
$\left|\sigma_{exp}-\sigma_{calc}\right|$	7.1	14.9	2.6	35.5		3.0

**Table 5:** The anisotropy coefficient for graphite foils produced by rolling and pressing.

	Specific Electrical Conductivity Anisotropy of the GF by Rolling (R)			Specific Electrical Conductivity Anisotropy of the GF by Pressing (P)
	RD/TD	RD/ND	TD/ND	<RD, TD>/ND
Experiment	1.09	158.63	146.09	120.99
Calculation	1.04	52.18	50.17	45.52

## Data Availability

The original contributions presented in this study are included in the article. Further inquiries can be directed to the corresponding author(s).
